# Genetic associations with healthy ageing among Chinese adults

**DOI:** 10.1038/s41514-022-00086-x

**Published:** 2022-05-02

**Authors:** Xuling Chang, Yan-Feng Zhou, Ling Wang, Jianjun Liu, Jian-Min Yuan, Chiea-Chuen Khor, Chew-Kiat Heng, An Pan, Woon-Puay Koh, Rajkumar Dorajoo

**Affiliations:** 1 Department of Paediatrics, Yong Loo Lin School of Medicine, National University of Singapore, Singapore, 119228 Singapore; 2 Khoo Teck Puat – National University Children’s Medical Institute, National University Health System, Singapore, 119074 Singapore; 3 Department of Epidemiology and Biostatistics, School of Public Health, Tongji Medical College, Huazhong University of Science and Technology, Wuhan, Hubei Province China; 4 Genome Institute of Singapore, Agency for Science, Technology and Research, Singapore, 138672 Singapore; 5 Department of Medicine, Yong Loo Lin School of Medicine, National University of Singapore, Singapore, 117597 Singapore; 6 Division of Cancer Control and Population Sciences, UPMC Hillman Cancer Center, University of Pittsburgh, Pittsburgh, PA 15232 USA; 7 Department of Epidemiology, Graduate School of Public Health, University of Pittsburgh, Pittsburgh, PA 15261 USA; 8 Singapore Eye Research Institute, Singapore National Eye Centre, Singapore, 169856 Singapore; 9 Ministry of Education Key Laboratory of Environment and Health, School of Public Health, Tongji Medical College, Huazhong University of Science and Technology, Wuhan, Hubei Province China; 10 State Key Laboratory of Environmental Health (Incubating), School of Public Health, Tongji Medical College, Huazhong University of Science and Technology, Wuhan, Hubei Province China; 11 Healthy Longevity Translational Research Programme, Yong Loo Lin School of Medicine, National University of Singapore, Singapore, 117545 Singapore; 12 Singapore Institute for Clinical Sciences, Agency for Science Technology and Research (A*STAR), Singapore, 117609 Singapore; 13 Health Services and Systems Research, Duke-NUS Medical School, Singapore, Singapore

**Keywords:** Genome, Quality of life, Epidemiology

## Abstract

The genetic basis of overall healthy ageing, especially among the East-Asian population is understudied. We conducted a genome-wide association study among 1618 Singapore Chinese elderly participants (65 years or older) ascertained to have aged healthily and compared their genome-wide genotypes to 6221 participants who did not age healthily, after a 20-year follow-up. Two genetic variants were identified (*P*_Meta_ < 2.59 × 10^−8^) to be associated with healthy aging, including the *LRP1B* locus previously associated in long-lived individuals without cognitive decline. Our study sheds additional insights on the genetic basis of healthy ageing.

Human life expectancy has increased remarkably over the last two centuries, however, this is often not translated as concomitant improvements in healthy life expectancy^1^. Genetic factors may influence longevity and survival to old age in good health (i.e. healthy ageing)^2^. Genome-wide association studies (GWAS) for longevity have been conducted in both Europeans and Asians, but only a few common genetic loci have been robustly replicated, such as the *APOE* locus^3,4^. This may be due to heterogeneity in the definition of long-lived cases and non-cases, as well as ethnic-specific genetic variabilities.

In addition to lifespan, healthy ageing is a complex outcome that reflects on the overall quality of life as well as cognitive and physical functions among the elderly population. Recent genetic studies for healthy ageing have primarily evaluated the incidence of major chronic diseases and defined healthy ageing as disease-free survival to old age^5–7^. Fewer studies have evaluated the multiple facets of healthy ageing in a complete manner, but nevertheless have described the important role of genetic factors in the process of ageing. A recent example is the identification of the *LRP1B* locus in long-lived individuals associated with both good cognitive function and the absence of major chronic diseases^8^. Nevertheless, holistic evaluations for healthy ageing have also been largely limited to studies performed in European ancestry populations^5,6,8^ and related data is sparse among participants of East-Asian ancestry.

The Singapore Chinese Health Study (SCHS) is a population-based prospective cohort study of Chinese adults living in Singapore^9^. Detailed information on the establishment of the cohort, genotyping methods and statistical analysis is available in the online supplementary documents. Seven aspects of healthy ageing in the SCHS was assessed by home visits at the third follow-up interview conducted between 2014 and 2016, and among participants who had survival to at least 65 years of age. Healthy ageing was defined as 1) absence of 10 major chronic diseases, 2) no evidence of cognitive impairment using education-specific cutoffs from Singapore-modified Mini-Mental State Examination (MMSE), 3) no limitations in instrumental activities of daily living (IADL) defined using the Lawton IADL scale, 4) no major depression defined as a score of less than 5 using the Geriatric Depression Scale (GDS), 5) good overall self-perceived health, 6) good physical functioning on self-report, and 7) no self-reported function-limiting pain. Using these seven domains to define healthy ageing has been widely used in the literature, and published previously in this Singapore Chinese cohort^10,11^.

A total of 7,839 study samples with genotyping data and who had information available to generate healthy ageing status were included in this study. The mean age of study participants at healthy ageing measurement was 73.36 (SD = 5.85) years and 40.72% were men (Supplementary Table 1). The proportion of individuals who met the criteria of healthy ageing was approximately 20% in this present genetic study (Supplementary Table 1), which was similar to the proportion in the large population that included those without genotyping data (*N* = 14,159)^10^. Compared to those that did not age healthily, participants who attained healthy ageing at follow-up 3 had lower BMI and lower proportions of current smokers and daily drinkers at baseline, but higher diet quality with higher aMED scores, and a higher proportion with secondary education and above at baseline (Supplementary Table 2). The study population was divided into a SCHS-discovery dataset (1,489 healthy ageing cases and 5,721 controls) and a SCHS-replication dataset (129 healthy ageing cases and 500 controls) according to the timing of the genotyping and the version of the Illumina Global Screening Array^12^. Associations between common variants [minor allele frequency (MAF) ≥ 1%] and healthy ageing were conducted in the discovery dataset and replication dataset, and meta-analysis was done subsequently using the inverse-variance method in a fixed-effect model. For identified hits, we further evaluated genetic associations with each of the seven components of healthy ageing in our dataset. Functional annotations of top hits were performed using Functional Mapping and Annotation (FUMA v1.3.6)^13^.

Two variants were associated with healthy ageing in our study (Table 1, Supplementary Fig. 1). *Rs138499810*, an intergenic single nucleotide polymorphism (SNP) on chromosome 5, was identified to be associated with healthy ageing at genome-wide significance levels in the discovery dataset and successfully validated in the replication dataset [T allele, odds ratio (OR) (95% confidence interval (Cl)] = 3.158 (2.148, 4.643), *P*_Meta_ = 4.94 × 10^−9^, Table 1, Supplementary Fig. 2a]. The association remained significant after adjustment for additional lifestyle-related variables, including BMI, dietary intake (aMED score), smoking status, alcohol consumption and education levels [T allele, odds ratio (OR) (95% confidence interval (Cl)] = 3.099 (2.110, 4.554), *P*_Meta_ = 8.21 × 10^−9^, Supplementary Table 3]. The frequency of the risk T allele for *rs138499810* was 2% in the SCHS study, which was similar to other 1000 Genomes East-Asian reference populations (i.e. CDX and CHB reference populations). In contrast, this SNP is monomorphic in most European ancestry populations, the exception being the 1000 Genomes Finnish reference population, which reported approximately 3% for this allele frequency^14^. Regional genes (*XRCC4*, *VCAN* and *TMEM167A*) within 10 kb of this variant were significantly enriched among loci identified for previous GWAS for biomarkers of osteoarthritis (Supplementary Fig. 3)^15^. Chromatin interaction mapping indicated that a significant interaction exists between the intergenic region containing *rs138499810* and the genes *XRCC4*, *VCAN* and *TMEM167A* in embryonic and mesenchymal stem cells (FDR between 3.37 × 10^−7^ and 1.99 × 10^−19^)^16^ (Supplementary Table 4). XRCC4 functions together with DNA ligase IV and the DNA-dependent protein kinase in the repair of DNA double-strand breaks. The efficiency of nonhomologous end joining (NHEJ) repair declines with age and restoration of XRCC4 improves NHEJ efficiency, suppresses the onset of stress-induced premature cellular senescence and mitigates ageing-related pathologies^17^. Additional studies would be warranted to unveil potential functional links between *rs138499810*, XRCC4 and ageing.

**Table 1:** Healthy ageing status association statistics of common genome-wide hits identified in the SCHS study.

						SCHS Discovery		SCHS Replication		Meta-analysis		
						Case = 1489 / Control = 5721		Case = 129 / Control = 500		Case = 1618 / Control = 6221		
	Chromosome	Position	Nearest gene	TA	TAF	OR (95% Cl)	*P*	OR (95% Cl)	*P*	OR (95% Cl)	*p*	p_Q_
rs138499810	5	82019798	Intergenic	T	0.020	3.104 (2.067, 4.663)	4.82 × 10^−8^	3.670 (1.103, 12.213)	0.034	3.158 (2.148, 4.643)	4.94 × 10^−9^	0.796
rs117898573	2	142877484	*LRP1B*	T	0.029	2.626 (1.888, 3.651)	9.65 × 10^−9^	1.218 (0.451, 3.291)	0.697	2.433 (1.779. 3.327)	2.59 × 10^−8^	0.151

*Chr* Chromosome, *TA* test allele, *TAF* test allele frequency, *OR* Odds ratio, *Cl* Confidence interval, *p*_*Q*_ Cochran’s Q heterogeneity *P* value.

The second variant identified was *rs117898573*. This was an East-Asian specific intronic variant at *LRP1B* on chromosome 2, and the minor allele, the T allele, had an allele frequency of 3% and the OR for the association with healthy ageing was 2.433 (95% CI 1.779, 3.327; *P*_Meta_ = 2.59 × 10^−8^) (Table 1, Supplementary Fig. 2b). Although we were unable to robustly replicate this association in the replication dataset, the direction of its effect in the replication dataset was consistent with that observed in the discovery analysis. Furthermore, the overall statistical significance still surpassed the genome-wide threshold level in the meta-analysis of all data. The effect estimates also remained similar after inclusion of additional lifestyle-related covariates in the association model [T allele, odds ratio (OR) (95% confidence interval (Cl)] = 2.327 (1.703, 3.179), *P*_Meta_ = 1.14 × 10^−7^, Supplementary Table 3]. The *LRP1B* locus has been associated with Alzheimer’s disease^18^, and haplotypes at *LRP1B* were previously reported to be associated with healthy ageing among long-lived individuals without cognitive decline^8^. Hence, our results corroborated the *LRP1B* gene locus for overall healthy ageing in the East-Asian population.

To unravel if the identified loci for overall healthy ageing may be associated with specific domains used to define the status of healthy ageing, we evaluated the associations between the identified SNPs and the individual components of healthy ageing in the SCHS dataset. *Rs138499810* was significantly associated with self-perceived health [OR (Cl) = 1.639 (1.226, 2.193), *P*_Adj_ = 7.81 × 10^−3^], while r*s117898573* was significantly associated with absence of function-limiting pain [OR (Cl) = 1.623 (1.212, 2.173), *P*_Adj_ = 0.010] and a null history of major chronic diseases [OR (Cl) = 1.451 (1.128, 1.866), *P*_Adj_ = 0.033] (Table 2).

**Table 2:** Association between healthy ageing associated genetic loci and 7 individual domains addressed by healthy ageing.

	rs138499810				rs117898573			
	TA	*P*	*P*_Adj_	OR (95% Cl)	TA	*P*	*P*_Adj_	OR (95% Cl)
No limitation in instrumental activities of daily living^1^	T	0.112	1.000	1.348 (0.933, 1.950)	T	0.670	1.000	1.067 (0.793, 1.435)
No function-limiting pain^2^		0.031	0.282	1.498 (1.037, 2.164)		0.001	0.010	1.623 (1.212, 2.173)
No impairment of cognitive function^3^		0.629	1.000	1.121 (0.704, 1.786)		0.062	0.562	1.406 (0.982, 2.012)
No clinical depression at screening^4^		0.155	1.000	1.275 (0.912, 1.782)		0.078	0.700	1.281 (0.973, 1.686)
No history of major chronic diseases^5^		0.014	0.127	1.463 (1.080, 1.983)		0.004	0.033	1.451 (1.128, 1.866)
Good physical function^6^		0.814	1.000	0.940 (0.560, 1.577)		0.130	1.000	1.351 (0.915, 1.994)
Good overall self-perceived health^7^		8.68 × 10^−4^	7.81 × 10^−3^	1.639 (1.226, 2.193)		0.051	0.455	1.270 (0.999, 1.614)

*TA* test allele, *OR* Odds ratio, *Cl* Confidence interval.

^1^Cases defined as IADL score >8.

^2^Cases defined as those who reported having no pain or discomfort.

^3^Cases defined as MMSE score ≥18 for participants with no formal education; ≥21 for primary school education; ≥25 for secondary school or higher.

^4^Cases defined as a GDS-15 score <5.

^5^Cases defined as no history of cancer, myocardial infarction, angina, heart failure, coronary artery bypass graft or angioplasty, stroke, diabetes, kidney failure, Parkinson’s disease, and chronic lung diseases.

^6^Cases defined as those who reported having no problem with mobility, self-care, and usual activities.

^7^Cases defined as those who rated their health as good or excellent.

Lastly, we evaluated if previous loci for longevity were associated with healthy ageing in this SCHS cohort. Interestingly, we found that the majority of the loci associated with increased longevity were not associated with healthy ageing in our study (Supplementary Table 5). However, it was consistent with the previous reports^5^ and indicated that most genetic determinants of longevity may be distinct from those involved in healthy ageing. Nevertheless, there were two longevity loci, *rs17514846* and *rs6108784*, which were nominally associated with healthy ageing in our study [*P* = 1.38 × 10^−4^ and *P* = 0.024, respectively, Supplementary Table 5]. The A allele in *rs17514846* and the C allele in *rs6108784* were previously associated with the phenotype of decreased longevity^19^, and these two alleles were observed with decreased healthy ageing in our study samples [OR (95% Cl) = 0.834 (0.746, 0.932) for the A allele in *rs17514846* and OR (95% Cl) = 0.911 (0.841, 0.988) for the C allele in *rs6108784*, Supplementary Table 5].

Our study identified genetic variants for overall healthy ageing in an East-Asian Chinese population. However, limitations need to be acknowledged. There is a concern that we were unable to significantly replicate the association of one variant, *rs117898573*, in our replication dataset, although the direction of effects at this SNP was consistent with the discovery data. This might be due to the relatively modest sample size of the replication dataset. Additionally, given the differences in definitions for healthy ageing, it would be challenging to evaluate the transferability of variants identified for related phenotypes, such as human health-span focused on disease-free survival to old age, which have been recently reported in European ancestry samples^6^. Furthermore, given that the variants identified in the present study were East-Asian specific, our data also highlights on the importance of potential ethnic-specific mechanisms that may influence healthy ageing in the population.

## Methods

### Study cohort

SCHS is a long-term population-based prospective cohort study focused on the dietary, genetic and environmental determinants of cancer and other chronic diseases in Singapore^9^. In brief, 63,257 subjects aged between 45 and 74 years were recruited from April 1993 to December 1998. The subjects belonged to two major Chinese dialect groups in Singapore (the Hokkien and the Cantonese). A total of 28,439 participants donated blood specimens. At recruitment, all the study subjects were interviewed in-person by an interviewer with a structured questionnaire. After recruitment, the participants were re-contacted for follow-up every 5 to 6 years. The in-person interview at the follow-up 3 was conducted from 2014 to 2016. Follow-up 3 was focused on the measurement of ageing outcomes and 17,107 surviving participants participated in this follow-up. The study was approved by the Institutional Review Board (IRB) of the National University of Singapore (NUS). Written informed consents were obtained from all study participants.

### Assessment of healthy ageing

The definition of healthy ageing in the SCHS has been previously described^20–22^. Healthy ageing addressed 7 domains, namely, no history of major chronic diseases, no impairment of cognitive function, no limitations in instrumental activities of daily living (IADL), no clinical depression at screening, good overall self-perceived health, good physical functioning and no function-limiting pain^10,23^. The major chronic diseases included in our definition of healthy ageing were compiled from previous studies^20–22^, which were asked in the baseline or subsequent follow-up questionnaires, and these, including cancer, myocardial infarction, angina, heart failure, coronary artery bypass graft or angioplasty, stroke, diabetes, kidney failure, Parkinson’s disease, and chronic lung diseases. The other components of healthy ageing were only assessed at the follow-up 3 visit cognitive function was evaluated using the Singapore-modified Mini-Mental State Examination (SM-MMSE), which had been validated in the Singapore population^24,25^; IADL was assessed on the basis of the Lawton IADL scale; clinical depression was screened using the 15-item geriatric depression scale (GDS-15), which had been validated in older Asians^26^; overall self-perceived health was assessed by asking a question “In general, would you say your health is: excellent, very good, good, fair, poor?”; physical function and function-limiting pain were assessed using the EuroQol Group’s 5-domain questionnaire (EQ-5D)^27^. Among participants who survived to at least age 65 years at the follow-up 3 visit, those who met these seven criteria were considered to have healthy ageing.

### Genotyping and Imputation

Twenty-seven thousand three hundred and eight SCHS samples were genotyped on the Illumina Global Screening Array (GSA). GWAS genotyping for 25,273 SCHS participants were completed in the year 2018 using the Illumina Global Screening v1.0 and v2.0 arrays and data were utilized as the SCHS Discovery in the present study. An independent set of 2035 SCHS participants were genotyped in the year 2020 using the Illumina Global Screening v2.0 and utilized in the present study as the SCHS Replication dataset. Detailed description for the quality control (QC) procedures has been previously described^28^. 7839 participants who had complete information to generate healthy ageing status and with overlapping genotypes were included in the current study. We imputed for additional autosomal SNPs using a two reference panel imputation approach by including both the cosmopolitan 1000 Genomes reference panels (Phase 3, 2,504 reference panels)^29^ and 4810 local Singapore Chinese (*N* = 2,780), Malays (*N* = 903), and Indians (*N* = 1,127) reference panels from a WGS study of the Singapore populations^30^. Alleles for all SNPs were coded to the forward strand and mapped to HG19. IMPUTE v2^31^ was used to mutually impute variants specific to 1000 Genomes and local Singaporean population sample reference panels into each other to obtain a merged reference panel for imputing untyped variants in study datasets. Imputation was performed in chunks of 1 Mb with a buffer of 500 kb and the effective size of the reference population, Ne, was set as 20,000 as recommended^31^. Frequencies of imputed common SNPs (MAF > 1%) showed strong correlation as compared to 1000 Genomes East Asian reference populations (EAS panels, R = 99.7%).

### Statistical method

Clinical characteristics for the study subjects at baseline were compared between healthy ageing cases and controls. Normally distributed quantitative traits, including body mass index (BMI) and alternate Mediterranean Diet Score (aMED score), were presented as mean ± SD (standard deviation) and the differences in means between cases and controls were compared by *t*-test. Categorical variables were presented as number of individuals and differences in their frequencies between groups were determined by Pearson’s χ2 test.

Genotype association analyses were done assuming an additive genetic model and with the inclusion of the—method score function in SNPTEST (version 2)^32^ to account of genotype uncertainties of imputed SNPs. To derive overall association values, META^33^ (version 1.5) was used to combine association summary statistics from discovery and replication datasets using the inverse-variance weighted method and assuming a fixed-effects model (*—*method 1). Heterogeneity of effects in meta-analyzed data was determined using Cochran’s Q and a Cochran’s Q *P*-value (P_het) < 0.05 was determined to be significantly heterogeneous. As lifestyle factors may affect the development of age-related diseases and health- and life-spans in the general population^34^, for identified hits, sensitivity analysis was done by including additional covariates in the regression model, namely body mass index (BMI, kg/m^2^), dietary intake (aMED score), smoking status (never, ever and current), alcohol consumption (nondrinker/monthly drinker, weekly drinker and daily drinker) and education levels (no formal education, primary school and secondary/A level/ University education) from baseline data. We additionally evaluated genetic associations between each of the seven components of healthy ageing and identified hits in our dataset.

Functional annotations for identified variants was performed using Functional Mapping and Annotation (FUMA v1.3.6)^13^. Datasets used were based on the HG19 human assembly. Variant MAF and LD (*r*^2^) were pre-computed using PLINK^35^ and based on the EAS 1000 Genomes reference population in FUMA. All SNPs in LD (*r*^2^ > 0.6 in 1000 G Phase 3 ASN panel) with lead SNPs from the study were identified. Regional genes (within 10 kb of lead SNP) at identified healthy ageing associated loci were mapped and annotated using the SNP2GENE function. Chromatin interaction mapping was analyzed as one end containing identified variants (region 1), that interacted with the 2nd region of the chromatin interactions that mapped to gene promoter regions (250 bp up- and 500 bp downstream of transcriptional start site by default) (region 2). Only significant interactions (FDR < 0.05) with mapped genes were retained for identified chromatin interactions. Gene set enrichment was done using the GENE2FUNC function in FUMA that evaluates for the enrichment of regional genes reported from the study, among putative GWAS catalogue genes from previous GWAS study results. Gene set enrichments with *P*_Adj_ < 0.05 were considered significant.

## Supplementary information

Supplemental information

healthy ageing_summary statistics

## Data Availability

Variants associated with healthy ageing status at *P* < 10^−5^ are available in Supplementary Table 6. Full meta-analysed data is available in Supplementary data.

## References

[1] Oeppen J. & Vaupel J. W. *Broken limits to life expectancy*. (American Association for the Advancement of Science, 2002). 10.1126/science.1069675 12004104

[2] Murabito JM, Yuan R, Lunetta KL (2012). The search for longevity and healthy aging genes: insights from epidemiological studies and samples of long-lived individuals. J Gerontol A Biol Sci Med Sci..

[3] Deelen J (2014). Genome-wide association meta-analysis of human longevity identifies a novel locus conferring survival beyond 90 years of age. Hum Mol Genet..

[4] Zeng Y (2016). Novel loci and pathways significantly associated with longevity. Sci Rep..

[5] Erikson GA (2016). Whole-Genome Sequencing of a Healthy Aging Cohort. Cell..

[6] Zenin A (2019). Identification of 12 genetic loci associated with human healthspan. Communications Biology..

[7] Walter S (2011). A genome-wide association study of aging. Neurobiol Aging..

[8] Poduslo S, Huang R, Spiro (2010). Iii A. A genome screen of successful aging without cognitive decline identifies LRP1B by haplotype analysis. American Journal of Medical Genetics Part B: Neuropsychiatric Genetics.

[9] Hankin JH (2001). Singapore Chinese Health Study: development, validation, and calibration of the quantitative food frequency questionnaire. Nutr Cancer.

[10] Zhou Y.-F. et al. Association between combined lifestyle factors and healthy ageing in Chinese adults: the Singapore Chinese Health Study. *J. Gerontol.: Series A*. **76**, 1796–1805 (2021). 10.1093/gerona/glab033 PMC8436980 33522576

[11] Zhou YF (2021). Association Between Dietary Patterns in Midlife and Healthy Ageing in Chinese Adults: The Singapore Chinese Health Study. J Am Med Dir Assoc.

[12] Dorajoo R (2019). Loci for human leukocyte telomere length in the Singaporean Chinese population and trans-ethnic genetic studies. Nat Commun..

[13] Watanabe K, Taskesen E, van Bochoven A, Posthuma D (2017). Functional mapping and annotation of genetic associations with FUMA. Nat Commun..

[14] Siva N (2008). 1000 Genomes project. Nat Biotechnol..

[15] Tachmazidou I (2019). Identification of new therapeutic targets for osteoarthritis through genome-wide analyses of UK Biobank data. Nat Genet..

[16] Fafián-Labora JA, Morente-López M, Arufe MC (2019). Effect of aging on behaviour of mesenchymal stem cells. World J Stem Cells..

[17] Li Z (2016). Impaired DNA double-strand break repair contributes to the age-associated rise of genomic instability in humans. Cell Death Differ..

[18] Shang Z (2015). Genome-wide haplotype association study identify TNFRSF1A, CASP7, LRP1B, CDH1 and TG genes associated with Alzheimer’s disease in Caribbean Hispanic individuals. Oncotarget..

[19] Pilling LC (2017). Human longevity: 25 genetic loci associated in 389,166 UK biobank participants. Aging (Albany N Y).

[20] Assmann KE (2018). Association between adherence to the Mediterranean diet at midlife and healthy aging in a cohort of French adults. J. Gerontol.: Series A..

[21] Sun Q (2011). Alcohol consumption at midlife and successful ageing in women: a prospective cohort analysis in the nurses’ health study. PLoS Med..

[22] Michel J-P, Sadana R (2017). “Healthy aging” concepts and measures. J Am Med Dir Assoc..

[23] Zhou Y.-F. et al. Association Between Dietary Patterns in Midlife and Healthy Ageing in Chinese Adults: The Singapore Chinese Health Study. *J. Am. Med. Dir. Assoc*. **22**, 1279–1286 (2020). 10.1016/j.jamda.2020.09.045 33218913

[24] Feng L, Chong MS, Lim WS, Ng TP (2012). The Modified Mini-Mental State Examination test: normative data for Singapore Chinese older adults and its performance in detecting early cognitive impairment. Singapore Med J..

[25] Katzman R (1988). A Chinese version of the Mini-Mental State Examination; impact of illiteracy in a Shanghai dementia survey. J Clin Epidemiol..

[26] Nyunt MSZ, Fones C, Niti M, Ng T-P (2009). Criterion-based validity and reliability of the Geriatric Depression Screening Scale (GDS-15) in a large validation sample of community-living Asian older adults. Aging and Mental Health.

[27] Group TE (1990). EuroQol-a new facility for the measurement of health-related quality of life. Health Policy.

[28] Chang X (2021). Low frequency variants associated with leukocyte telomere length in the Singapore Chinese population. Communications biology.

[29] Auton A (2015). A global reference for human genetic variation. Nature..

[30] Wu D (2019). Large-Scale Whole-Genome Sequencing of Three Diverse Asian Populations in Singapore. Cell..

[31] Marchini J, Howie B, Myers S, McVean G, Donnelly P (2007). A new multipoint method for genome-wide association studies by imputation of genotypes. Nat Genet..

[32] Marchini J, Howie B, Myers S, McVean G, Donnelly P (2007). A new multipoint method for genome-wide association studies by imputation of genotypes. Nat Genet..

[33] Liu JZ (2010). Meta-analysis and imputation refines the association of 15q25 with smoking quantity. Nat Genet..

[34] Brooks-Wilson AR (2013). Genetics of healthy aging and longevity. Hum Genet..

[35] Purcell S (2007). PLINK: a tool set for whole-genome association and population-based linkage analyses. Am. J. Human Genet..

