# Supplementary material for: Genetic associations with healthy ageing among Chinese adults
Source: NPJ Aging. 2022 May 2;8(1):6. doi: 10.1038/s41514-022-00086-x (PMC9158790; doi:10.1038/s41514-022-00086-x)

Supplementary table 1. Characteristics of study subjects in SCHS.

	SCHS Discovery N = 7,210	SCHS replication N = 629	SCHS entire dataset N = 14,159
Healthy ageing status	1,489 (20.65%)	129 (20.51%)	2,834 (20.02%)
No limitation in instrumental activities of daily living[1]	5,415 (75.10%)	485 (77.11%)	10,403 (73.47%)
No function-limiting pain[2]	5,794 (80.36%)	497 (79.01%)	11,427 (80.71%)
No impairment of cognitive function[3]	6,487 (88.95%)	592 (91.08%)	12,108 (85.52%)
No clinical depression at screening[4]	5,404 (74.95%)	454 (72.18%)	10,451 (73.81%)
No history of major chronic diseases[5]	4,706 (65.27%)	434 (69.00%)	9,247 (65.31%)
Good physical functioning[6]	6,406 (88.85%)	568 (90.30%)	12,454 (87.96%)
Good overall self-perceived health[7]	3,618 (50.18%)	306 (48.65%)	7,012 (49.52%)
Age (years)	73.39 ± 5.87	73.09 ± 5.68	73.70 ± 6.03
sex (% males)	2,978 (41.30%)	214 (34.02%)	5,808 (41.02%)

Data was presented as mean ± standard deviation or N (percentage).

1. Cases defined as IADL score > 8.

2. Cases defined as those who reported having no pain or discomfort.

3. Cases defined as MMSE score ≥18 for participants with no formal education; ≥21 for primary school education; ≥25 for secondary school or higher.

4. Cases defined as a GDS-15 score <5.

5. Cases defined as no history of cancer, myocardial infarction, angina, heart failure, coronary artery bypass graft or angioplasty, stroke, diabetes, kidney failure, Parkinson's disease, and chronic lung diseases.

6. Cases defined as those who reported having no problem with mobility, self-care, and usual activities.

7. Cases defined as those who rated their health as good or excellent.

Supplementary table 2. Comparison of baseline lifestyle factors for heathy ageing cases and controls in the study.

	SCHS Discovery			SCHS Replication			SCHS entire dataset		
	Healthy ageing cases	Controls		Healthy ageing cases	Controls		Healthy ageing cases	Controls	
N	1,489	Control = 5,721	P	129	500	P	2,834	11,325	P
BMI (kg/m^2)	22.57 ± 2.79	23.26 ± 3.20	<0.001	22.23 ± 2.92	22.99 ± 3.41	0.012	22.50 ± 2.87	23.24 ± 3.27	<0.001
alternate Mediterranean Diet Score	4.50 ± 1.67	4.21 ± 1.64	<0.001	4.55 ± 1.70	4.26 ± 1.66	0.083	4.46 ± 1.67	4.15 ± 1.67	<0.001
Smoking status									
never smokers	1,229 (82.54%)	4,387 (76.68%)		110 (85.27%)	421 (84.20%)		2,295 (80.98%)	8,689 (76.72%)	
ever smokers	114 (7.66%)	521 (9.11%)	<0.001	15 (11.63%)	22 (4.40%)	<0.001	228 (8.05%)	1,020 (9.01%)	<0.001
current smokers	146 (9.81%)	813 (14.21%)		4 (3.10%)	57 (11.40%)		311 (10.97%)	1,616 (14.27%)	
Alcohol consumption									
nondrinker/monthly drinker	1,306 (87.71%)	5,067 (88.57%)		111 (86.05%)	454 (90.80%)		2,467 (87.05%)	10,062 (88.85%)	
weekly drinker	152 (10.21%)	491 (8.58%)	0.045	15 (11.63%)	33 (6.60%)	0.158	293 (10.34%)	936 (8.26%)	0.002
daily drinker	31 (2.08%)	163 (2.85%)		3 (2.33%)	13 (2.60%)		74 (2.61%)	327 (2.89%)	
Education levels									
no formal education	195 (13.10%)	1,025 (17.92%)		15 (11.63%)	97 (19.40%)		401 (14.15%)	2,380 (21.02%)	
primary school	624 (41.91%)	2,666 (46.60%)	<0.001	60 (46.51%)	222 (44.40%)	0.108	1,194 (42.13%)	5,272 (46.55%)	<0.001
secondary/A level/ University education	670 (45.00%)	2,030 (35.48%)		54 (41.86%)	181 (36.20%)		1,239 (43.72%)	3,673 (32.43%)	

Data was presented as mean ± SD or N (%).

Supplementary table 3. Healthy ageing status associations for genome-wide hits identified in the SCHS study after further adjustment for additional lifestyle related variables, including baseline BMI, dietary intake (aMED score), smoking status, alcohol consumption and education levels .

						SCHS Discovery Case = 1,489 / Control = 5,721		SCHS Replication Case = 129 / Control = 500		Meta-analysis Case = 1,618 / Control = 6,221		
	Chromosome	Position	Nearest gene	TA	TAF	OR (95% CI)	P	OR (95% CI)	P	OR (95% CI)	P	P_Q
rs138499810	5	82019798	Intergenic	T	0.020	3.083 (2.052, 4.632)	5.97×10^{-8}	3.240 (1.003, 10.467)	0.049	3.099 (2.110, 4.554)	8.21×10^{-9}	0.938
rs117898573	2	142877484	*LRP1B*	T	0.029	2.494 (1.796, 3.463)	4.86×10^{-8}	1.207 (0.440, 3.314)	0.715	2.327 (1.703, 3.179)	1.14×10^{-7}	0.180

Chr: Chromosome; TA: test allele; TAF: test allele frequency; OR: Odds ratio; CI: Confidence interval; $P_{Q:}$ Cochran's Q heterogeneity Pvalue.

Supplementary table 4. Significant 3D chromatin interaction mapping identified for rs138499810.

region1	region2	FDR	type	DB	tissue/cell	SNPs	genes
5:82000001-82040000	5:82360001-82400000	1.99E-19	HiC	GSE87112	Mesenchymal_Stem_Cell	rs138499810	*TMEM167A:XRCC4*
5:82000001-82040000	5:82760001-82800000	8.24E-11	HiC	GSE87112	Mesenchymal_Stem_Cell	rs138499810	*VCAN*
5:82000001-82040000	5:82360001-82400000	1.66E-17	HiC	GSE87112	Mesendoderm	rs138499810	*TMEM167A:XRCC4*
5:82000001-82040000	5:82760001-82800000	1.38E-15	HiC	GSE87112	Mesendoderm	rs138499810	*VCAN*
5:82000001-82040000	5:82360001-82400000	1.16E-13	HiC	GSE87112	hESC	rs138499810	*TMEM167A:XRCC4*
5:82000001-82040000	5:82760001-82800000	3.37E-07	HiC	GSE87112	hESC	rs138499810	*VCAN*

Supplementary table 5. Summary association statistics of known longevity associated genetic variants with healthy ageing status in SCHS.								
SNP	chromosome	position	Gene	RA	TA	OR (95% CI)	P	Reference study
rs146254978	1	74867799	*FPGT / TNNI3K*			Monomorphic in EAS		Pilling, L. C., et al. (2017)
rs602633	1	109821511	CLESR2; PSRC1	T	G	0.944 (0.800, 1.113)	0.494	Pilling, L. C., et al. (2017)
rs1425609	3	162681995	*OTOL1*	G	A	1.030 (0.927, 1.145)	0.581	Murabito, J. M., et al. (2012)
rs10491334	5	110772404	*CAMKIV*	C	T	0.920 (0.758, 1.116)	0.395	Murabito, J. M., et al. (2012)
rs3130507	6	31147476	*PSORS1C3*	G	A	1.059 (0.962, 1.167)	0.240	Pilling, L. C., et al. (2017)
rs3131621	6	31425499	*MICA; MICB*	A	G	1.078 (0.932, 1.247)	0.313	Pilling, L. C., et al. (2017)
rs28383322	6	32592796	*HLA-DRB1… HLA-DQA1*	C	T	1.079 (0.957, 1.218)	0.215	Pilling, L. C., et al. (2017)
rs1627804	6	107400428	*BEND3*	C	A	1.037 (0.956, 1.126)	0.379	Pilling, L. C., et al. (2017)
rs2802292	6	108908518	*FOXO3a*	G	T	0.960 (0.882, 1.045)	0.342	Murabito, J. M., et al. (2012)
rs1935949	6	108999287	*FOXO3A*	A	G	0.936 (0.858, 1.022)	0.139	Erikson, G. A., et al. (2016)
rs55730499	6	161005610	*LPA*			Monomorphic in EAS		Pilling, L. C., et al. (2017)
rs2069837	7	22768027	*IL6*	A	G	0.946 (0.853, 1.050)	0.300	Zeng, Y., et al. (2016)
rs7844965	8	27442064	*EPHX2*	G	A	1.088 (0.975, 1.215)	0.130	Pilling, L. C., et al. (2017)
rs13262617	8	59838133	*TOX*			Monomorphic in EAS		Pilling, L. C., et al. (2017)
rs2811712	9	21998035	*CDKN2A*	G	A	1.084 (0.985, 1.194)	0.099	Erikson, G. A., et al. (2016)
rs1556516	9	22100176	*CDKN2B-AS1 (ANRIL)*	G	C	0.936 (0.861, 1.017)	0.119	Pilling, L. C., et al. (2017)
rs3758391	10	69643342	*SIRT1*	T	C	1.046 (0.932, 1.174)	0.444	Erikson, G. A., et al. (2016)
rs9664222	10	89338633	*MINPPI*	A	C	1.008 (0.910, 1.116)	0.885	Murabito, J. M., et al. (2012)
rs61905747	11	113639842	*ZW10*			Monomorphic in EAS		Pilling, L. C., et al. (2017)
rs2542052	11	116699984	*APOC3*	A	C	1.022 (0.944, 1.107)	0.593	Erikson, G. A., et al. (2016)
rs139137459	11	119269958	*USP2-AS1*			Monomorphic in EAS		Pilling, L. C., et al. (2017)
rs7137828	12	111932800	*SH2B3 / ATXN2*			Monomorphic in EAS		Pilling, L. C., et al. (2017)
rs1043332229	13	31871514	*B3GALTL*			Monomorphic in EAS		Pilling, L. C., et al. (2017)
rs61949650	13	64836488	*Intergenic*	T	C	0.969 (0.802, 1.171)	0.744	Pilling, L. C., et al. (2017)
rs61978928	14	75321714	*PROX2*	T	C	1.053 (0.958, 1.157)	0.288	Pilling, L. C., et al. (2017)
rs3803304	14	105239146	*AKT1*	C	G	1.058 (0.911, 1.230)	0.459	Erikson, G. A., et al. (2016)
rs74011415	15	47660194	*SEMA6D*	G	A	1.023 (0.901, 1.162)	0.725	Pilling, L. C., et al. (2017)
rs1317286	15	78896129	*CHRNA3*	A	G	0.945 (0.833, 1.073)	0.384	Pilling, L. C., et al. (2017)
rs17514846	15	91416550	*FURIN*	C	A	0.834 (0.746, 0.932)	1.38×10^{-4}	Pilling, L. C., et al. (2017)
rs34516635	15	99451976	*IGF1R*			Monomorphic in EAS		Erikson, G. A., et al. (2016)
rs5882	16	57016092	*CETP*	G	A	1.030 (0.952, 1.115)	0.459	Erikson, G. A., et al. (2016)
rs1042522	17	7579472	*TP53*	G	C	1.026 (0.949, 1.110)	0.516	Erikson, G. A., et al. (2016)
rs28926173	18	13886719	*MC2R*			Monomorphic in EAS		Pilling, L. C., et al. (2017)
rs12461964	19	41341229	*EGLN2; CYP2A6*			Monomorphic in SCHS		Pilling, L. C., et al. (2017)
rs429358	19	45411941	*APOE / APOC1*	T	C	0.935 (0.811, 1.078)	0.354	Pilling, L. C., et al. (2017)
rs4420638	19	45422946	*APOC1*	A	G	0.925 (0.813, 1.052)	0.233	Murabito, J. M., et al. (2012)
rs2075650	19	45395619	*TOMM40*	A	G	0.923 (0.802, 1.063)	0.268	Murabito, J. M., et al. (2012)
rs74444983	19	45745607	*EXOC3L2; MARK4*			Monomorphic in EAS		Pilling, L. C., et al. (2017)
ApoE	19				E4 allele	0.938 (0.810, 1.086)	0.391	Murabito, J. M., et al. (2012)
rs6108784	20	10964366	*C20orf187*	T	C	0.911 (0.841, 0.988)	0.024	Pilling, L. C., et al. (2017)
rs2273500	20	61986949	*CHRNA4*	T	C	0.961 (0.852, 1.083)	0.515	Pilling, L. C., et al. (2017)

RA: reference allele; TA: test allele; TAF: test allele frequency; OR: odds ratio; CI: confidence interval.

Supplementary table 6. Sub-GWAS significant variants ($P < 10^{-5}$) for healthy ageing status in SCHS.								
chr	rsid	pos	allele_A	allele_B	P_value	beta	se	P_heterogeneity
5	rs1384998	82019798	C	T	4.94E-09	1.150	0.197	0.796
2	rs1178985	142877484	C	T	2.59E-08	0.889	0.160	0.151
2	rs1394620	142885483	A	T	3.66E-08	1.026	0.186	0.162
7	rs1169890	9898368	T	C	6.07E-08	0.586	0.108	0.823
7	rs1413471	13448225	G	T	7.47E-08	1.304	0.242	0.116
8	rs1365690	125952148	T	C	1.65E-06	-0.314	0.065	0.181
8	rs7813655	125935058	C	G	1.73E-06	-0.301	0.063	0.293
8	rs7833872	125932332	A	G	1.77E-06	-0.301	0.063	0.251
3	rs5588566	5125012	A	G	2.05E-06	0.260	0.055	0.917
7	rs3733781	13311414	A	C	2.10E-06	0.967	0.204	0.120
3	rs3711223	87365227	T	A	2.22E-06	1.047	0.221	0.042
5	rs7876904	144424330	C	T	2.35E-06	0.414	0.088	0.153
7	rs3776936	13294122	A	G	2.79E-06	0.951	0.203	0.121
5	rs7673813	144383882	C	T	2.92E-06	0.403	0.086	0.119
11	rs1918542	94075295	A	G	3.00E-06	0.799	0.171	0.204
5	rs1170181	144457274	G	A	3.00E-06	0.451	0.097	0.325
17	rs6208373	56266494	A	C	3.07E-06	0.726	0.156	0.974
8	rs7004579	125953439	T	C	3.16E-06	-0.311	0.067	0.190
10	rs7895403	13154110	A	G	3.60E-06	0.199	0.043	0.687
8	rs7463584	125926827	G	A	3.63E-06	-0.290	0.063	0.396
8	rs6470325	125928974	A	G	3.73E-06	-0.289	0.062	0.390
14	rs1711035	26229620	C	T	3.77E-06	0.237	0.051	0.945
5	rs1430525	144410985	C	T	4.47E-06	0.402	0.088	0.114
2	rs1884969	205390408	G	C	4.56E-06	1.344	0.293	0.842
12	rs2845009	41804869	A	T	4.67E-06	0.202	0.044	0.456
11	rs3413781	99580124	T	G	4.86E-06	0.206	0.045	0.805
8	rs2881078	125914333	T	C	4.87E-06	-0.291	0.064	0.468
3	rs9845957	5126325	T	C	4.99E-06	0.248	0.054	0.780
11	rs3587660	99580057	A	C	5.18E-06	0.205	0.045	0.809
3	rs5615640	5135199	G	A	5.32E-06	0.214	0.047	0.497
11	rs1713421	99566782	A	G	5.46E-06	0.204	0.045	0.831
19	rs1492516	58827877	A	G	5.55E-06	0.403	0.089	0.540
7	rs1885665	13462136	T	C	5.67E-06	1.014	0.223	0.335
9	rs7985670	117355308	A	C	5.70E-06	0.777	0.171	0.692
11	rs1434718	94163420	C	T	5.80E-06	0.765	0.169	0.289
11	rs3441952	99565867	G	A	5.82E-06	0.203	0.045	0.827
1	rs3540897	19180369	C	T	5.87E-06	0.335	0.074	0.884
3	rs1389145	13198934	C	T	5.94E-06	0.992	0.219	0.577
11	rs3457201	99581336	G	C	5.97E-06	0.204	0.045	0.741
11	rs1241947	99565102	T	A	6.17E-06	0.203	0.045	0.828
10	rs2806453	134942319	C	T	7.14E-06	0.946	0.211	0.379
12	rs1473269	25480598	G	C	7.39E-06	0.919	0.205	0.083
3	rs9883556	5136038	G	C	7.61E-06	0.212	0.047	0.573
14	rs7746688	50572807	C	T	7.76E-06	0.576	0.129	0.449
11	rs2002356	99545042	C	A	7.81E-06	0.194	0.043	0.415
6	rs1175843	67295955	C	T	8.66E-06	1.038	0.233	0.102
20	rs1903935	4941761	T	C	8.93E-06	-0.466	0.105	0.365

Supplementary Figure 1. Genome-wide healthy ageing associations in the SCHS meta-analysis (1,618 Case/ 6,221 Non-cases). **a.** 2 loci at chromosome 2 and 5 were identified beyond the genome-wide significance threshold (*P* < 5.0 × 10^{-8}, red line). **b.** QQ-plot of observed compared to expected P-values indicated minimal inflation of study results (λ = 1.0004).

a **b**

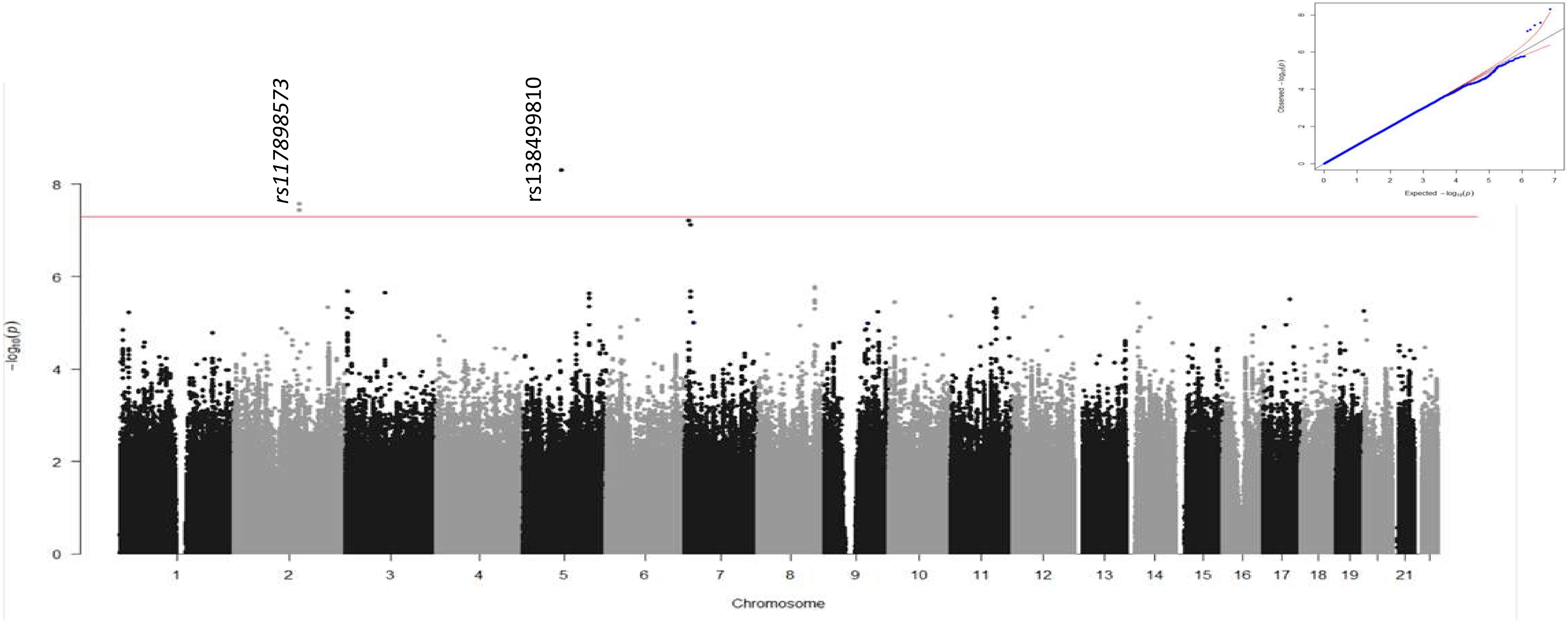

Supplementary Figure 2. Regional common SNP associations at **a.** Intergenic (rs138499810, chr5) and **b.** *LRP1B* (rs117898573, chr2) regions. Plots plotted using LocusZoom (http://csg.sph.umich.edu/locuszoom/).

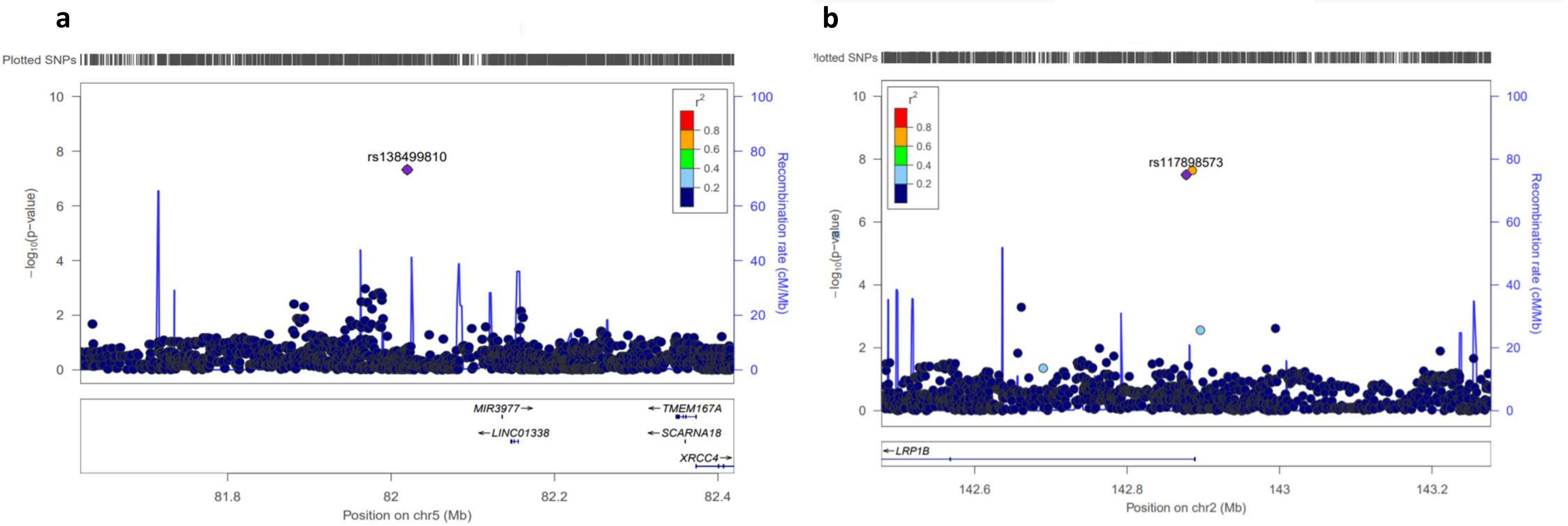

Supplementary Figure 3: Enrichment of regional genes at identified hits for healthy ageing status in the SCHS study among previous GWAS data.

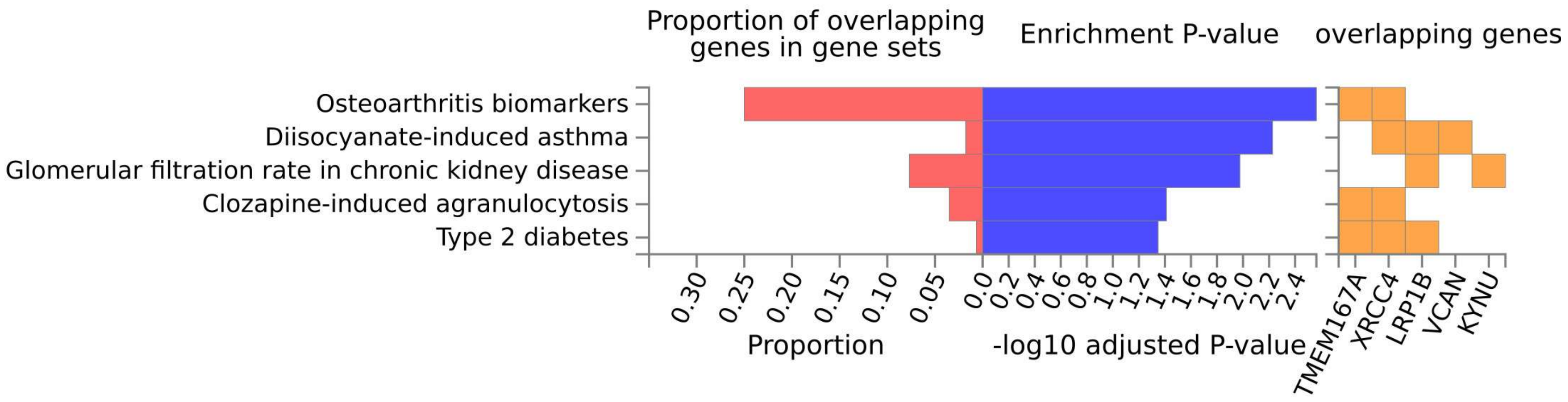

Supplement: Supplementary file 1 — Supplemental information [file 41514_2022_86_MOESM1_ESM.pdf]
